# Interleukin-22 and interleukin-33 show up-regulated levels in the serum of patients with mild/moderate Coronavirus disease 2019

**DOI:** 10.1186/s43088-023-00367-8

**Published:** 2023-02-26

**Authors:** Abdulraheem Y. Majeed, Nor Effa S. Zulkafli, Ali H. Ad’hiah

**Affiliations:** 1 Department of Biomedical Sciences, Advanced Medical and Dental Institute, Universiti Sains Malaysia, Bertam, 13200 Penang, Malaysia; 2 Tropical-Biological Research Unit, College of Science, University of Baghdad, Al-Jadriya, Al-Karrada, 10070 Baghdad, Iraq

**Keywords:** COVID-19, IL-22, IL-33, Inflammation

## Abstract

**Background:**

This study analyzed serum concentrations of interleukin (IL)-22 and IL-33 (pro-inflammatory and anti-inflammatory cytokines) in 90 patients with mild/moderate coronavirus disease 2019 (COVID-19) and 90 healthy controls. Enzyme-linked immunosorbent assay kits were used to measure IL-22 and IL-33 concentrations.

**Results:**

Median (interquartile range) concentrations of IL-22 and IL-33 were significantly higher in patients than in controls (IL-22: 18.6 [18.0–19.3] *vs*. 13.9 [12.1–14.9] pg/mL, probability [*p*] < 0.001; IL-33: 37.8 [35.3–43.0] *vs*. 24.1 [23.0–26.2] pg/mL, *p* < 0.001). As indicated by the area under the curve (AUC), IL-22 and IL-33 were excellent predictors of COVID-19 (AUC = 0.95 and 0.892, respectively). Multinomial logistic regression analysis demonstrated that individuals with high production (> control median) of IL-22 (odds ratio = 17.80 [95% CI: 6.48–48.90]; *p* = 0.001) and IL-33 (odds ratio = 19.0 [95% CI: 7.4–48.6]; *p* = 0.001) were more likely to develop COVID-19. A positive correlation was found between IL-22 and IL-33 and both cytokines also showed positive correlations with granulocyte-to-lymphocyte ratio and erythrocyte sedimentation rate in all participants.

**Conclusions:**

IL-22 and IL-33 showed up-regulated concentrations in the serum of patients with mild/moderate COVID-19. Both cytokines may have prognostic value for COVID-19 along with their association with disease risk.

## Background

During the past two years, humanity has faced a global challenge due to a serious respiratory viral infection, specifically the Coronavirus Disease 2019 (COVID-19). This disease has caused high morbidity and mortality rates worldwide. The causative agent is a highly pathogenic RNA virus belonging to the Coronaviridae family (severe acute respiratory syndrome coronavirus-2; SARS-CoV-2) [1]. Initial symptoms include headache, dry cough, difficulty breathing and fever, but in severe/serious illness, complications of COVID-19 may progress to acute respiratory distress syndrome (ARDS) and consequent multi-organ failure and death [2]. Studies have indicated that the pathogenic mechanisms involved in COVID-19 are primarily related to a dysregulated immune response. In this context, the term hypercytokinemia has been applied to describe the dysregulated production of cytokines and other immunological markers in COVID-19 patients [3].

Cytokines are low-molecular-weight glycoproteins produced by both immune and non-immune cells to mediate cellular interactions and regulate immune responses against various pathogens. With regard to inflammation, they are broadly categorized into pro-inflammatory and anti-inflammatory cytokines, and recent evidence suggests that a balance between the two types of cytokines is of functional importance in any immune response [4]. In COVID-19, it is becoming clear that cytokines with pro-inflammatory functions play a fundamental role in the pathogenesis of the disease, particularly associated complications such as ARDS [5]. Most COVID-19 patients develop mild/moderate illness, while some people infected with SARS-CoV-2 may develop hyper-inflammation resulting from overproduction of pro-inflammatory cytokines, which are a major cause of fatal pneumonia and ARDS [6]. Tumor necrosis factor (TNF)-α, interleukin (IL)-1, IL-6, interferon (IFN)-γ, IL-17A and IL-8 are among the pro-inflammatory cytokines that have been mostly studied in patients with COVID-19. Elevated circulating levels of these cytokines have been linked to the pathogenesis of COVID-19, as well as clinical manifestations of severe disease [7, 8]. IL-22 and IL-33 are among the least explored pro-inflammatory cytokines in COVID-19. Recent evidence indicates that both cytokines may have a role in the pathogenesis and/or severity of COVID-19 [9–13]. Although IL-22 and IL-33 are recognized as pro-inflammatory cytokines, they also have anti-inflammatory functions [14, 15]. Therefore, exploring IL-22 and IL-33 in COVID-19 and their relationship to each other may expand our understanding of the pathogenic mechanism associated with the disease.

Most previous studies were concerned with examining systemic concentrations of IL-22 and IL-33 in patients with severe/critical COVID-19, while data for mild/moderate cases are not overwhelming. Therefore, the current study aimed to analyze serum IL-22 and IL-33 concentrations in non-hospitalized patients exposed to mild/moderate COVID-19. The correlations between IL-22 and IL-33 and with other inflammatory markers in patients and controls were also analyzed.

## Methods

### Subjects

A study was conducted on 90 non-hospitalized patients who were exposed to mild/moderate COVID-19 during January-October 2021. Patients were referred to health care units affiliated to hospitals in Al-Anbar Province (100 km north of the capital, Baghdad) due to signs and symptoms of the emerging COVID-19. On admission, a nasopharyngeal swab was obtained and molecularly tested for SARS-CoV-2 RNA (RealLine SARS-CoV-2 kit, Bioron Diagnostics GmbH). Besides, SARS-CoV-2 antibodies (IgM/ IgG) were tested in the serum of patients (VIDAS SARS-CoV-2 IgM/ IgG assay kits; bioMerieux, France). The World Health Organization (WHO) interim guidance was followed to determine the severity of COVID-19 [16]. Inclusion criteria included a positive molecular test, a positive IgM antibody test, and age 18 years and over. In addition, only patients with mild/moderate COVID-19 were enrolled [16]. Pregnant women were excluded. Ninety volunteers were included and considered as a control group (HC). They were apparently healthy individuals (blood donors), and their serum tests for SARS-CoV-2 IgM, IgG and antibodies to other viruses (anti-HIV, anti-HCV, and HBsAg) were negative (data on these antibodies was provided by the Al-Anbar Province Blood Bank). Approval to conduct the study was obtained from the Ethics Committee of the Al-Anbar Directorate of Health, Iraqi Ministry of Health (Approval No. 2022027). All subjects agreed to participate in the study and provided a written consent.

### Baseline data of subjects

Data pertaining to age, sex and body mass index (BMI) were documented for each participant. In addition, the following laboratory tests were performed: white blood cell (WBC), granulocyte and lymphocyte counts, erythrocyte sedimentation rate (ESR), liver function tests (alanine transaminase [ALT], aspartate aminotransferase [AST] and alkaline phosphatase [ALP]), lipid profile (total cholesterol and triglycerides) and random blood glucose (RBG). Granulocyte-to-lymphocyte ratio (GLR) and lymphocyte-to-monocyte ratio (LMR) were calculated as previously described [17].

### Laboratory methods

Seven to nine days post-SARS-CoV-2- infection, 10 mL of blood was obtained and processed for laboratory tests required for the study. Enzyme-linked immunosorbent assay (ELISA) kits were used to measure serum IL-22 and IL-33 concentrations (Catalogue number: 900-M246 and 900-M398, respectively; PeproTech EC Ltd, UK) following the manufacturer's instructions. ESR was assessed using the conventional Westergren method. Complete blood count was performed using CELL-DYN Automated Emerald Hematology Analyzer (Abbott, USA). Serum ALT, AST, ALP, total cholesterol, triglyceride, and RBG concentrations were assessed using BIOLIS 30i analyzer preloaded with the respective kit reagents (Tokyo Boeki, Japan).

### Statistical analysis

Data were given as number and percentage (categorical variable), mean and standard deviation (SD; parametric variable) or median and interquartile range (IQR; non-parametric variable). Significant differences were assessed using Pearson's Chi-square, one-way analysis of variance (ANOVA) or Mann–Whitney *U* test, respectively. The prognostic value of each cytokine was evaluated using receiver operating characteristic (ROC) curve analysis and presented in terms of the area under the curve (AUC) and its 95% confidence interval (CI). Besides, cut-off point, sensitivity and specificity were also estimated. The Youden index (YI) was used to optimize the cut-off point. The odds ratio (OR) and 95% CI were calculated using multinomial regression analysis (adjusted for age and sex). In this analysis, participants were classified into two groups, low-production (LP; ≤ median) and high-production (HP; > median), based on the median concentration of each cytokine in the HC group. Correlation coefficient (r_s_) was determined using the non-parametric Spearman's rank correlation analysis. The 0.05 level (probability; *p*) was taken statistically significant after adjustment for multiple comparisons (Bonferroni correction). GraphPad Prism version 8.0.0 (San Diego, CA, USA) and IBM SPSS Statistics 25.0 (Armonk, NY: IBM Corp.) were employed to carry out statistical analysis. G*power 3.1.9.7 was used to estimate the sample size power.

## Results

### Sample size power

The sample size power of 90 patients with COVID-19 and 90 HC was analyzed using the G*power software. The analysis was conducted with 0.05 two-tailed alpha-error *p* and 0.5 effect size d. The estimated sample size power was 0.91. The statistically validated power of sample size is 0.8 [18].

### Baseline data

**Fig. 1:**
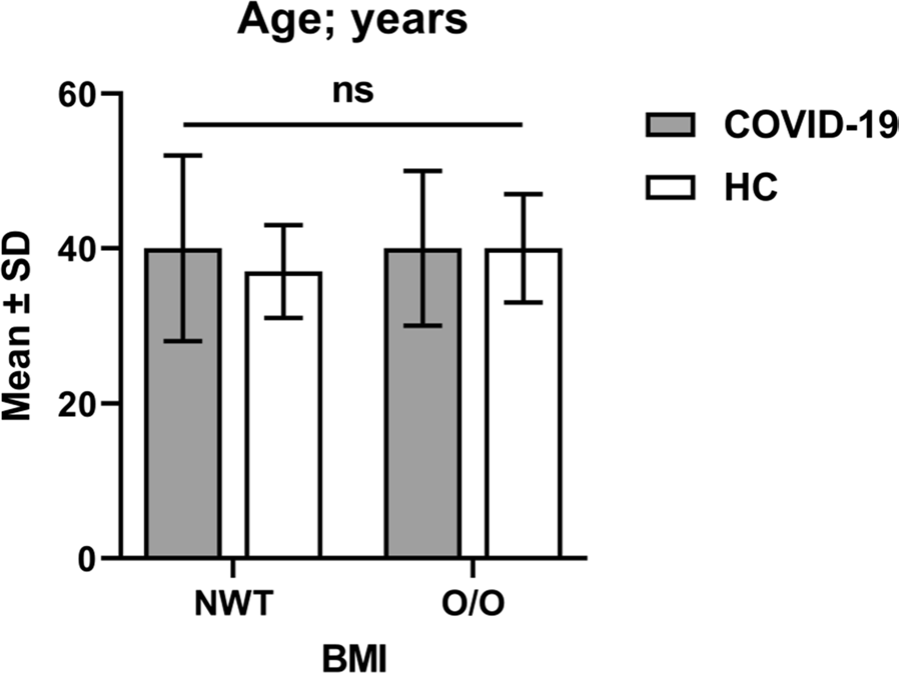
Column-bar plot of age in COVID-19 patients and healthy controls (HC) stratified by body mass index (BMI). Columns represent mean. Bars represent standard deviation (SD). Significant difference was assessed using one-way analysis of variance (ns: *p*-value is not significant; > 0.05). The mean age was 40 ± 12 and 37 ± 6 years in normal-weight individuals (NWT) and 40 ± 10 and 40 ± 7 years in overweight/obese (O/O) individuals from COVID-19 patients and HC, respectively. The difference between the means was not significant (*p* = 0.423)

As shown in Table 1, the COVID-19 patients and HC were matched in terms of age (40.0 ± 10.5 *vs*. 38.9 ± 6.6 years; *p* = 0.423), age group (20–39 years: 48.9 *vs*. 53.3%; 40–65 years: 51.1 *vs.* 46.7%; *p* = 0.551), sex (male: 60.0 *vs*. 64.4%; female: 40.0 *vs*. 35.6%; *p* = 0.539), and BMI (normal-weight: 23.3 *vs*. 31.1%; overweight/obese: 76.7 *vs*. 68.9%; *p* = 0.241). In addition, when the age of patients and HC was stratified by BMI, there was no significant difference either (*p* = 0.423) (Fig. 1). The SARS-CoV-2 antibody test showed that all patients were negative for IgG and positive for IgM. The IgG titer was 0.24 ± 0.21 U/mL (< 1.0 U/mL) and IgM titer was 1.64 ± 0.35 U/mL (> 1.0 U/mL). ESR was significantly increased in patients compared with HC (46.3 ± 17.2 *vs*. 13.5 ± 4.5 mm/h; *p* < 0.001). Significantly elevated counts of WBCs (8.5 ± 3.1 *vs*. 7.0 ± 1.3 × 10^9^/L; *p* < 0.001) and granulocytes (5.9 ± 2.7 *vs*. 3.9 ± 1.1 × 10^9^/L; *p* < 0.001) were observed in patients compared with HC, while lymphocyte count was significantly decreased in patients (2.1 ± 0.9 *vs*. 2.6 ± 0.6 × 10^9^/L; *p* < 0.001). Regardless of these differences, the three counts of blood cells were almost within the normal range. Monocyte count did not show a significant variation between patients and HC (*p* = 0.118). GLR was significantly higher in patients than in HC (3.4 ± 2.2 *vs*. 1.6 ± 0.5; *p* < 0.001), while LMR was significantly lower in patients (3.6 ± 1.2 *vs*. 4.9 ± 1.5; *p* < 0.001). Serum concentrations of ALT, AST, ALP, and cholesterol did not show a significant variation between patients and HC (*p* = 0.161, 0.21, 0.09 and 0.827, respectively). On the contrary, triglycerides and RBG (131.3 ± 32.1 *vs*. 117.0 ± 32.6 mg/dL; *p* = 0.004 and 153.6 ± 85.4 vs. 99.1 ± 16.3 mg/dL; *p* < 0.001, respectively) showed significantly elevated concentrations in patients compared with HC (Table 1).

**Table 1:** Baseline data for COVID-19 cases and controls

Variable^a^		COVID-19 cases; *n* = 90	HC; *n* = 90	*p*-value
Age; year		40.0 ± 10.5	38.9 ± 6.6	0.423
Age group; year	20–39	44 (48.9)	48 (53.3)	0.551
	40–65	46 (51.1)	42 (46.7)	
Gender	Male	54 (60.0)	58 (64.4)	0.539
	Female	36 (40.0)	32 (35.6)	
BMI	Normal-weight	21 (23.3)	28 (31.1)	0.241
	Overweight/obese	69 (76.7)	62 (68.9)	
SARS-CoV-2 IgG titer; U/L	0.24 ± 0.21	NA	NA	
SARS-CoV-2 IgM titer; U/L	1.64 ± 0.35	NA	NA	
ESR; mm/h	46.3 ± 17.2	13.5 ± 4.5	** < 0.001**	
WBC × 10^9^/L	8.5 ± 3.1	7.0 ± 1.3	** < 0.001**	
Granulocyte × 10^9^/L	5.9 ± 2.7	3.9 ± 1.1	** < 0.001**	
Lymphocyte × 10^9^/L	2.1 ± 0.9	2.6 ± 0.6	** < 0.001**	
Monocyte × 10^9^/L	0.6 ± 0.3	0.5 ± 0.1	0.118	
GLR	3.4 ± 2.2	1.6 ± 0.5	** < 0.001**	
LMR	3.6 ± 1.2	4.9 ± 1.5	** < 0.001**	
ALT; U/L	37.4 ± 33.0	32.1 ± 13.0	0.161	
AST; U/L	30.7 ± 13.2	28.7 ± 7.2	0.21	
ALP; IU/L	91.6 ± 57.0	80.6 ± 22.2	0.09	
Cholesterol; mg/dL	188.3 ± 102.3	182.9 ± 20.3	0.827	
Triglycerides; mg/dL	131.3 ± 32.1	117.0 ± 32.6	**0.004**	
RBG; mg/dL	153.6 ± 85.4	99.1 ± 16.3	** < 0.001**	

*HC* Healthy controls, *BMI* Body mass index, *SARS-CoV-2* Severe acute respiratory syndrome coronavirus-2, *ESR* Erythrocyte sedimentation rate, *WBC* White blood cell, *GLR* Granulocyte-to-lymphocyte ratio, *LMR* Lymphocyte-to-monocyte ratio, *ALT* Alanine transaminase, *AST* Aspartate aminotransferase; ALP: Alkaline phosphatase; RBG: Random blood glucose, *p*: Two-tailed probability (significant *p*-value is indicated in bold), *NA* Not applicable

^a^Data are given as mean ± standard deviation (parametric variable) or number followed by percentage in parenthesis (categorical variable). Significance was determined using one-way analysis of variance test (parametric variable) or Pearson's Chi-square test (categorical variable)

### IL-22 and IL-33 concentrations

**Fig. 2:**
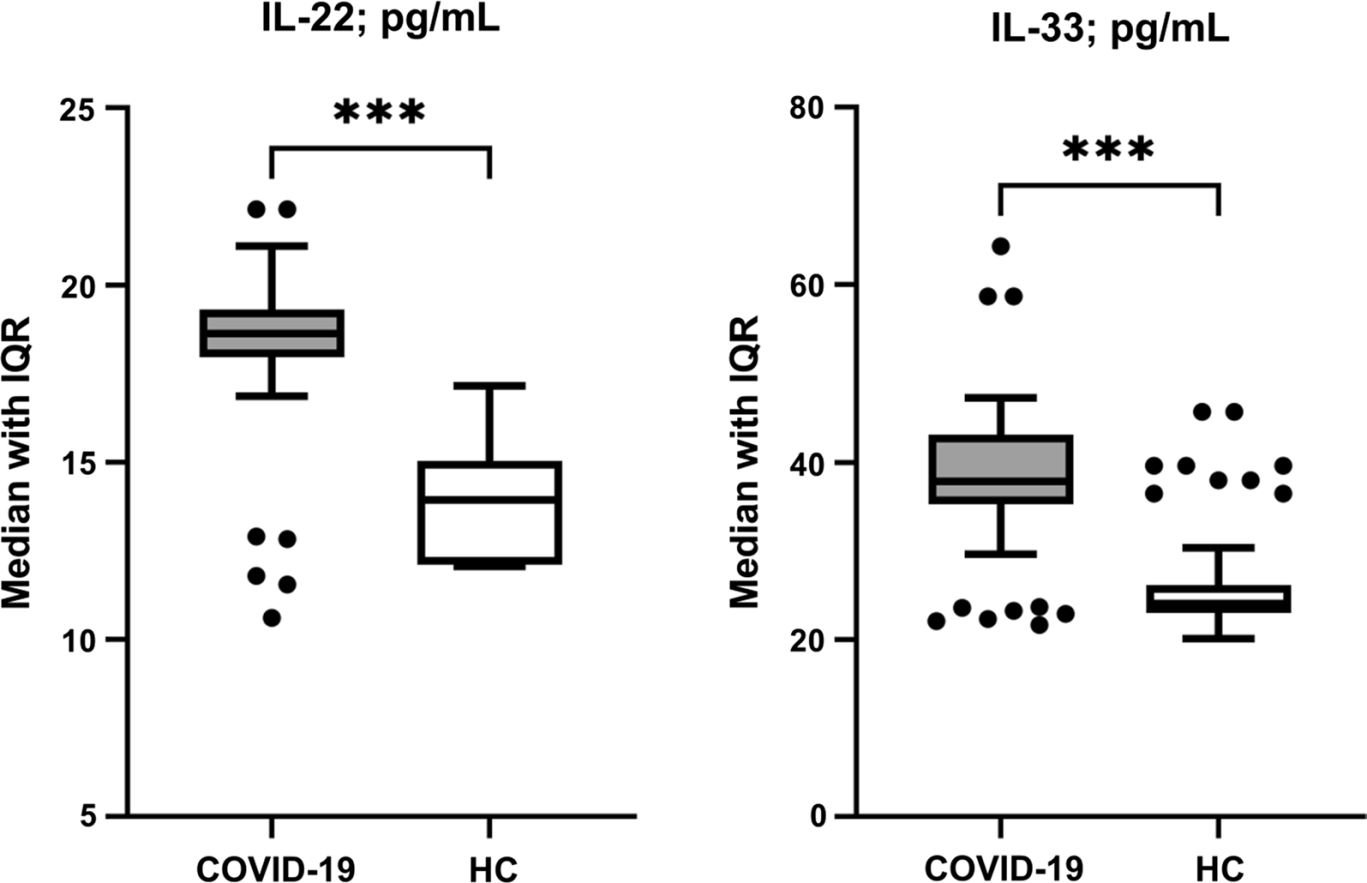
Box and whisker plots (Tukey method) of serum concentrations of IL-22 and IL-33 in COVID-19 patients (*n* = 90) and healthy controls (HC; *n* = 90). Horizontal line inside box represents median. Whiskers represent interquartile range (IQR). Black circles represent outliers. Significant difference was assessed using Mann–Whitney *U* test (***p < 0.001). Median IL-22 and IL-33 concentrations were significantly elevated in COVID-19 patients compared with HC (IL-22: 18.6 [IQR: 18.0–19.3] *vs*. 13.9 [IQR: 12.1–14.9] pg/mL, *p* < 0.001); IL-33: 37.8 [IQR: 35.3–43.0] *vs*. 24.1 [IQR: 23.0–26.2] pg/mL, *p* < 0.001)

Median IL-22 and IL-33 concentrations were significantly elevated in COVID-19 patients compared with HC (IL-22: 18.6 [IQR: 18.0–19.3] *vs*. 13.9 [IQR: 12.1–14.9] pg/mL, *p* < 0.001); IL-33: 37.8 [IQR: 35.3–43.0] *vs*. 24.1 [IQR: 23.0–26.2] pg/mL, *p* < 0.001) (Fig. 2). When IL-22 and IL-33 concentrations were stratified according to age group (18–39 *vs*. 40–65 years), sex (male *vs*. female) or BMI (normal-weight *vs*. overweight/obese) in COVID-19 patients, only normal-weight patients showed significantly higher IL-22 concentrations than in overweight/obese patients (19.2 [IQR: 18.1–19.5] *vs*. 18.5 [IQR: 17.9–19.2] pg/mL; *p* = 0.04). When the stratification of IL-22 and IL-33 concentrations was conducted in HC or all participants (patients plus HC), no significant difference was found in each stratum (Table 2).

**Table 2:** Serum interleukin (IL-22 and IL-33) concentrations stratified by age group, gender, and body mass index in COVID-19 cases, controls, and all participants (patients plus controls)

Group	Median (IQR); pg/mL					
	COVID-19; n = 90		Controls; n = 90		All; n = 180	
	IL-22	IL-33	IL-22	IL-33	IL-22	IL-33
*Age; year*						
20–39	18.9 (18.0–19.3)	40.1 (36.3–43.3)	13.9 (12.7–14.9)	24.0 (23.0–28.0)	16.8 (13.8–18.8)	31.4 (23.5–40.2)
40–65	18.5 (18.0–19.2)	37.7 (34.9–42.0)	13.9 (12.1–15.5)	24.1 (23.0–25.9)	17.0 (13.7–18.5)	30.7 (23.9–39.3)
*p*-value	0.422	0.379	0.913	0.974	0.938	0.911
*Gender*						
Male	18.5 (18.0–19.2)	39.3 (36.4–43.5)	14.1 (12.7–14.9)	24.0 (23.0–26.2)	16.6 (13.8–18.4)	29.8 (23.7–39.6)
Female	19.1 (18.1–19.5)	37.4 (35.3–42.1)	13.8 (12.1–15.2)	24.1 (22.6–26.1)	17.2 (13.5–19.1)	33.0 (24.1–39.9)
*p*-value	0.141	0.352	0.585	0.797	0.518	0.558
*BMI*						
NWT	19.2 (18.1–19.5)	39.5 (34.9–43.4)	13.7 (12.4–15.2)	25.1 (23.3–28.9)	16.3 (13.5–19.1)	29.8 (24.1–38.0)
O/O	18.5 (17.9–19.2)	37.8 (36.2–42.7)	14.1 (12.1–14.9)	23.8 (22.1–25.8)	17.2 (14.1–18.5)	32.4 (23.7–39.7)
*p*-value	**0.04**	0.981	0.517	0.072	0.868	0.874

*IL* Interleukin, *IQR* Interquartile range, *BMI* Body mass index, *NWT* Normal-weight; *O/O* Overweight/obese, *p* Two-tailed probability (significant *p*-value is indicated in bold). Mann–Whitney *U* test was used to assess significant differences between medians

### ROC curve analysis

**Fig. 3:**
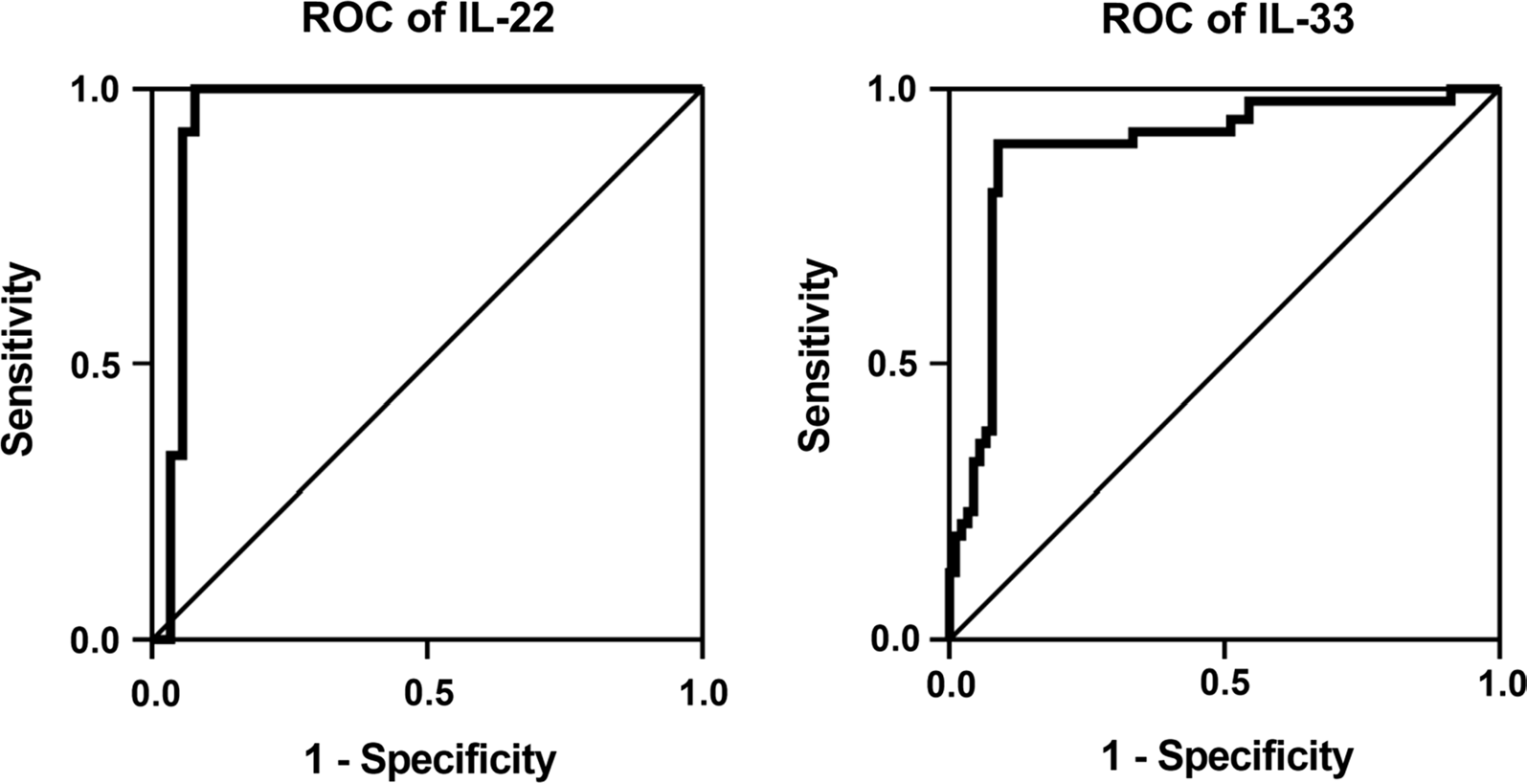
Reciever operating characteristic (ROC) curve analysis of IL-22 and IL-33 in COVID-19 cases *versus* healthy controls. IL-22 (area under the curve [AUC] = 0.95; 95% confidence interval [CI] = 0.908–0.992; *p* < 0.001; cut-off point = 17.3 pg/mL; Youden index [YI] = 0.88; sensitivity = 95.9%; specificity = 92.2%) and IL-33 (AUC = 0.892; 95% CI = 0.839–0.945; *p* < 0.001; cut-off point = 30.7 pg/mL; YI = 0.8; sensitivity = 90.0%; specificity = 90.0%) showed excellent prognostic value in differentiating patients from controls. The AUC is estimated through ROC analysis to describe the overall performance of a biomarker (i.e. cytokine serum level) to discriminate between cases with and without the disease under study. In general, the AUC value follows the following scheme: 0.50–0.59 (no discrimination), 0.60–0.69 (poor discrimination), 0.70–0.79 (acceptable), 0.80–0.89 (excellent) and ≥ 0.9 (outstanding)

As indicated by the AUC, ROC curve analysis indicated the potential of IL-22 (AUC = 0.95; 95% CI = 0.908–0.992; *p* < 0.001; cut-off point = 17.3 pg/mL; YI = 0.88; sensitivity = 95.9%; specificity = 92.2%) and IL-33 (AUC = 0.892; 95% CI = 0.839–0.945; *p* < 0.001; cut-off point = 30.7 pg/mL; YI = 0.8; sensitivity = 90.0%; specificity = 90.0%) in differentiating COVID-19 patients from HC (Fig. 3).

### Multinomial logistic regression analysis

Multinomial logistic regression analysis was performed to assess the significance of IL-22 and IL-33 as factors associated with COVID-19 risk. Each participant was coded as LP or HP for each cytokine based on the median concentration in HC (≤ and > median, respectively). The OR was estimated for HP *versus* LP. Higher concentrations of IL-22 and IL-33 were associated with an increased risk of COVID-19 (IL-22: OR = 17.80 [95% CI: 6.48–48.90], *p* = 0.001); IL-33: OR = 19.0 [95% CI: 7.4–48.6], *p* = 0.001) (Table 3).

**Table 3:** Age- and gender-adjusted multinomial logistic regression analysis of the association between interleukins (IL-22 and IL-33) and COVID-19

Cytokine; pg/mL	Group	Cases; n = 90		HC; n = 90		OR (95% CI)	*p*-value (*pc*)
		n	%	n	%		
IL-22	LP	5	5.6	45	50.0	Reference (1.0)	** < 0.001 (0.001)**
	HP	85	94.4	45	50.0	17.80 (6.48–48.90)	
IL-33	LP	7	7.8	51	56.7	Reference (1.0)	** < 0.001 (0.001)**
	HP	83	92.2	39	43.3	19.0 (7.4–48.6)	

*IL* Interleukin, *LP* Low-production (≤ control median), *HP* High-production (> control median), *HC* Healthy controls, *OR* Odds ratio, *CI* Confidence interval, *p*: Two-tailed probability, *pc*: Bonferroni correction probability (significant *p*-value is indicated in bold)

### Correlation analysis

**Fig. 4:**
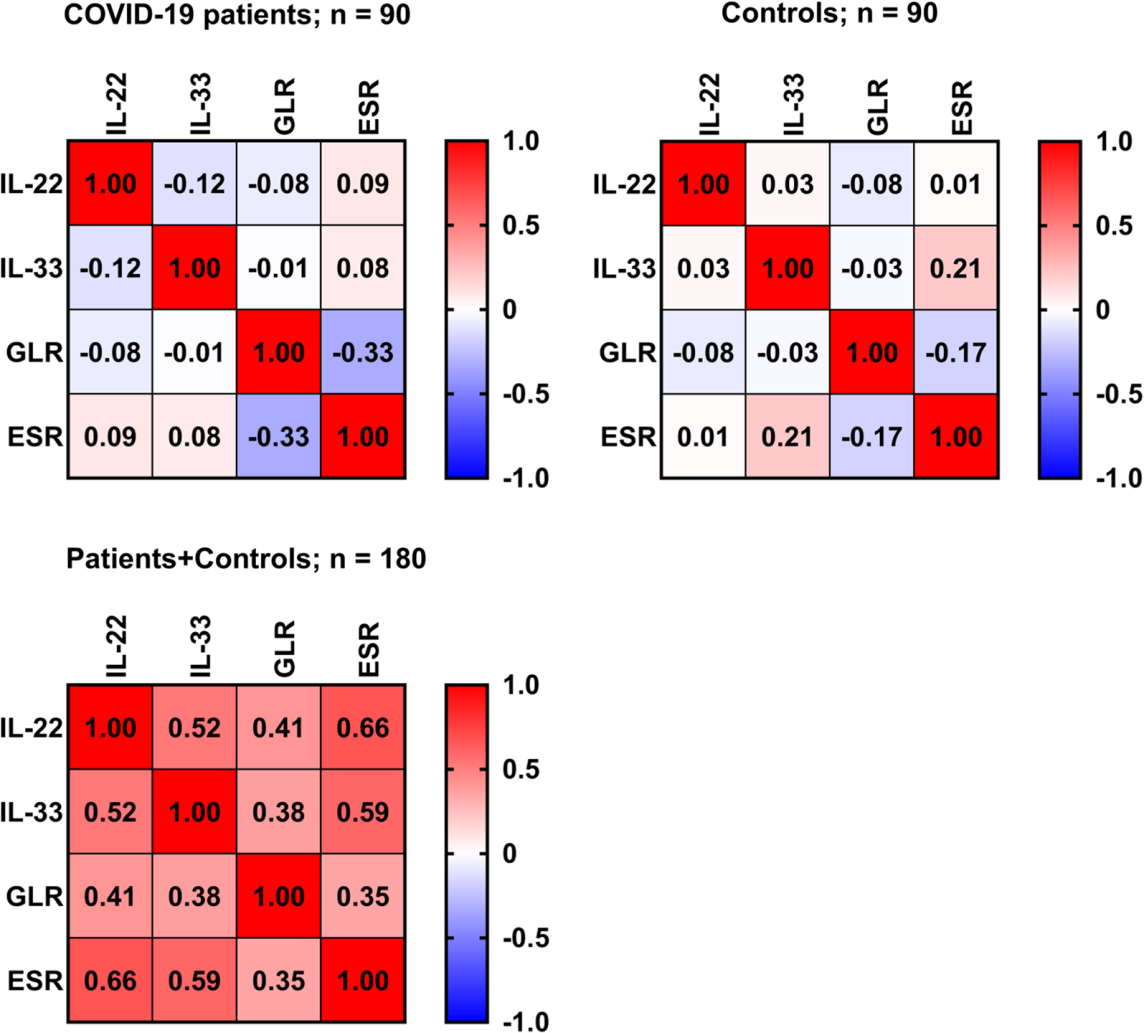
Heat-map matrix of correlation (Spearman's rank correlation analysis) between IL-22, IL-33, granulocyte-to-lymphocyte ratio (GLR), and erythrocyte sedimentation rate (ESR) among COVID- 19 patients only, healthy controls (HC) only, and patients plus HC. Values inside boxes indicate the correlation coefficient (r_s_). Red color indicates a positive correlation, while blue color indicates a negative correlation. IL-22 and IL-33 showed no significant correlation in COVID-19 patients (r_s_ =  − 0.12; *p* = 0.258) or HC (r_s_ = 0.03; *p* = 0.775). In addition, both cytokines showed no significant correlation with GLR and ESR in patients or HC. IL-33 and ESR were an exception and showed significant positive correlation in HC (r_s_ = 0.21; *p* = 0.049). GLR negatively correlated with ESR in patients (r_s_ =  − 0.33) and HC (r_s_ =  − 0.17), but the correlation was only significant in patients (*p* = 0.001). However, the pattern of correlations was different when the analysis was performed in all participants (COVID-19 patients plus HC). IL-22 positively correlated with IL-33 (r_s_ = 0.52; *p* < 0.001), GLR (r_s_ = 0.41; *p* < 0.001), and ESR (r_s_ = 0.66; *p* < 0.001). IL-33 positively correlated with GLR (r_s_ = 0.38; *p* < 0.001) and ESR (r_s_ = 0.59; *p* < 0.001). Finally, a positive correlation between GLR and ESR was found (r_s_ = 0.35; *p* < 0.001)

Spearman's rank correlation analysis was performed between IL-22, IL-33, GLR, and ESR for all possible combinations. IL-22 and IL-33 showed no significant correlation in COVID-19 patients (r_s_ = − 0.12; *p* = 0.258) or HC (r_s_ = 0.03; *p* = 0.775). In addition, both cytokines showed no significant correlation with GLR and ESR in patients or HC. IL-33 and ESR were an exception and showed significant positive correlation in HC (r_s_ = 0.21; *p* = 0.049). GLR negatively correlated with ESR in patients (r_s_ = − 0.33) and HC (r_s_ = − 0.17), but the correlation was only significant in patients (*p* = 0.001). However, the pattern of correlations was different when the analysis was performed in all participants (COVID-19 patients plus HC). IL-22 positively correlated with IL-33 (r_s_ = 0.52; *p* < 0.001), GLR (r_s_ = 0.41; *p* < 0.001), and ESR (r_s_ = 0.66; *p* < 0.001). IL-33 positively correlated with GLR (r_s_ = 0.38; *p* < 0.001) and ESR (r_s_ = 0.59; *p* < 0.001). Finally, a positive correlation between GLR and ESR was found (r_s_ = 0.35; *p* < 0.001) (Fig. 4).

## Discussion

In this study, serum IL-22 and IL-33 concentrations were analyzed in patients with mild/moderate COVID-19 and compared with age-, sex- and BMI-matched HC. Patients showed significantly up-regulated concentrations of IL-22 and IL-33. IL-22, a newly described member of the IL-10 cytokine family, is produced largely by a distinct subset of CD4 + T cells called T helper (h) 22. This cytokine has been pointed out to exert a number of immunomodulatory effects with the potential to perform both pro-inflammatory and anti-inflammatory functions [19]. Regarding pro-inflammatory effects, observations in mice and humans revealed that IL-22 coordinates the inflammatory response by affecting the synthesis of other pro-inflammatory cytokines, including IL-6, TNF-α, and IL-8. Besides, IL-22 promotes the release of the pro-inflammatory cytokine IL-1β in a caspase 1-dependent manner [14, 19]. IL-22 has also been associated with pro-inflammatory activity in several inflammatory diseases such as psoriasis, asthma, ankylosing spondylitis, and atopic dermatitis [20–22]. On the other hand, anti-inflammatory effects of IL-22 have been identified in other inflammatory conditions such as inflammatory bowel disease [14, 19, 23]. In response to microbial infection, IL-22 has been found to be a potent inducer of the inflammatory response, especially in viral infections, such as hepatitis B, C and HIV [24].

The current study revealed that up-regulated concentrations of IL-22 were an excellent predictor of COVID-19 (AUC = 0.95). Also, individuals with elevated production of IL-22 have a 17.80 -fold higher risk of disease. IL-22 has not been well explored in the immunopathogenesis of COVID-19 as few studies have been conducted in this context. Consistent with our observation, IL-22 showed up-regulated serum levels in COVID-19 patients < 16 years of age [12]. In addition, evidence has been provided that IL-22 receptor 1 (IL22R1) shows abnormal expression in blood myeloid cells and CD4 + T cells during SARS-CoV-2 infection [25]. Accordingly, manipulation of this cytokine has been proposed as a potential immunotherapy for the effective management of COVID-19 [9].

IL-33 is the next cytokine that showed up-regulated concentrations in COVID-19 patients, very good discrimination potential between patients and HC (AUC = 0.892), and a 19.0-fold increased risk of disease. IL-33 is a cytokine of the IL-1 family potentiated with pro-inflammatory and anti-inflammatory effects in response to infection [15]. Infected alveolar epithelial cells are the main source of IL-33 in the lungs, and activated SARS-CoV-2-derived papain-like protease can efficiently promote these cells to secrete IL-33, particularly in patients with severe COVID-19 [13]. However, the potential role of IL-33 in the immunopathogenesis of COVID-19 has not been well detailed, but it is suggested that IL-33 plays a fundamental role in lung homeostasis [11]. It has been demonstrated that serum IL-33 concentrations were significantly increased in patients with severe/critical COVID-19 compared to patients with mild/moderate disease, but all severe/critical patients were over 64.5 years old and 81.8% were male. The authors concluded that up-regulated IL-33 levels may facilitate lung inflammation by promoting the production of a range of innate pro-inflammatory cytokines, including TNF-α, IL-1β, IL-6, IL-12, IL-23 [10]. These findings pointed out that up-regulated IL-33 levels may be associated with SARS-CoV-2 infection and indicate that IL-33 may play a role in the immunopathogenesis of COVID-19.

Correlation analysis revealed no significant relationship between IL-22 and IL-33 in COVID-19 patients or HC. The correlation with GLR and ESR was also not clear. This is probably due to the low sample size of patients and HC. To avoid the effect of sample size, Spearman's correlation analysis was performed in all participants (patients plus HC). This time, the pattern of correlations between IL-22, IL-33, GLR, and ESR was different and the four biomarkers positively correlated. It has been well documented that ESR and GLR are inflammatory markers associated with the pathogenesis of COVID-19 [26], and IL-22 and IL-33 may represent additional inflammatory markers associated with the development of mild/moderate SARS-CoV-2 infection.

Our study encountered some limitations. First, despite an acceptable power estimate of the sample size, a larger cohort of patients and controls is preferred. Second, patients were not evaluated for studied cytokines and other laboratory variables at first referral to health care units. Third, other pro-inflammatory cytokines, as well as anti-inflammatory cytokines, should be evaluated simultaneously to understand their functional relationship, particularly during SARS-CoV-2 infection and convalescence.

## Conclusions

IL-22 and IL-33 showed up-regulated serum concentrations in patients with mild/moderate COVID-19. Both cytokines may have prognostic value for COVID-19 along with their association with disease risk.

## Data Availability

The datasets used and/or analyzed during the current study are available from the corresponding author on reasonable request.

## Abbreviations

ALP: Alkaline phosphatase
ALT: Alanine transaminase
ARDS: Acute respiratory distress syndrome
AST: Aspartate aminotransferase
AUC: Area under the curve
CI: Confidence interval (CI)
COVID-19: Coronavirus disease 2019
ESR: Erythrocyte sedimentation rate
GLR: Granulocyte-to-lymphocyte ratio
HC: Healthy controls
HP: High-production
IFN: Interferon
IL: Interleukin
IQR: Interquartile range
LMR: Lymphocyte-to-monocyte ratio
LP: Low-production
NWT: Normal-weight
O/O: Overweight/obese
OR: Odds ratio
*p*: Probability
*pc*: Corrected probability
RBG: Random blood glucose (RBG)
ROC: Receiver operating characteristic
r_s_: Correlation coefficient
SARS-CoV-2: Severe acute respiratory syndrome coronavirus-2
TNF: Tumor necrosis factor
WBC: White blood cell
YI: Youden index (YI)
